# Some Cases in Conservative Gynecology

**Published:** 1900-02

**Authors:** G. Betton Massey

**Affiliations:** Philadelphia, Pa.


### SOME CASES IN CONSERVATIVE GYNECOLOGY.*

By G. BETTON MASSEY, M.D.,

PHILADELPHIA, PA.

That there has been a reaction against a conception in gynecology which concerned itself only with the removal of one or more of the pelvic organs of woman all will admit. Our best operators appear, one after the other, with papers bearing a general appearance of conservatism, and we are told with much detail how a portion of an ovary or a portion of fibromatous uterus may be left in place, or how a diseased uterus or tube may be removed through the vagina. This is well, so far as it goes, and comes with a good

*Read at the Philadelphia County Medical Society, December 27, 1899.

grace from men who, only five or six years ago, were writing papers on "My Second Hundred Ovariotomies" or "A Year's Work in Abdominal Section," and incidentally saying nothing of the blasted hopes and blasted lives left after their work. That medical men should exhibit this great change of front in so short a time speaks volumes for the self-purging progress of a single generation. The older fad that every patient should be bled lasted hundreds of years, and though it looked as if every woman would loose her ovaries several years ago, the comparatively quick restoration of reason in the matter is a most hopeful augury of future medical progress.

But progress is at times greater on the surface than beneath. We want esoteric progress as well as exoteric progress. We want, in other words, fewer surgeons with a private reputation for removing the ovaries of every woman brought to them.

Of the value of those procedures in conservative gynecology which require cutting operations it is not my province to speak beyond the expression of an opinion that they represent progress in the right direction. By an equally just division of the responsibility flowing only from experience, I am empowered to speak definitely of certain other lines of conservative work in which I am personally engaged, and also to compare them with the results of still other lines of conservative work that have been employed in the cases coming under my observation.

It is self-evident that it is the prime duty of the physician to cure his patient, but it should be no less a prime duty to accomplish this result with least risk of harm to the patient, and to conserve all of her functions that are compatible with health and comfort.

Gynecology is a new specialty, and there has been much to and fro marching of its master minds. In the present stage of its progress it would be wise to emulate the example of the merchant, and once a year "take an account of stock" of its remedial procedures. In such an examination all will agree that major surgery, in this aseptic age, is the only possible recourse in the cure of ovarian and dermoid tumors and purulent collections; and that minor surgery is a blessing in those plastic operations in which so much true skill and dexterity are required. Aside from malignant conditions, which will not be further mentioned in this paper, and fibroid tumors, which will be referred to later, a critical study will show that the great majority of the remaining affections peculiar to women are more or less connected with inflammation or infection, whether the exciting cause be traumatic or not.

It is for one or other of this great sub- or post-inflammatory group that most patients consult physicians, though it is to be regretted that medical men too often overlook this evident pathologic fact while concentrating their attention on mere mechanical results. When this broader conception of these conditions prevails the profession will condemn unsparingly the removal of ovaries for mere inflammation or adhesion, or even prolapse; and as it pronounces the judgment that this too frequently performed operation has been due to culpable carelessness, it will also say that the removal of the uterus for the same conditions was culpable stupidity.

The sharp curette in the treatment of endometritis has been unsparingly condemned by many surgeons, yet I hear of its use every day. The enlightened consensus of present opinion demands that the use of this instrument be stopped, even if more troublesome procedures are thereby made necessary for the attainment of results.

The steel dilator is a barbarous instrument to employ in a cervix which is not physiologically prepared to dilate, as when post-partum débris is to be removed, and as its use in the normally rigid cervix often leads to periuterine inflammation, other and safer means should be used in its place when possible.

Of pessaries, I may say that I have not placed one in position for years, except in cases of strictly senile relaxation, and on the contrary making quite a collection of these crutches in my office, and in no case has the patient regretted the loss of this incubus, being invariably made well by removing inflammatory sequels, reducing the size of the uterus and toning up the ligaments.

By what means, then, can we cure these cases preventing post-inflammatory, catarrhal and congestive conditions?

I need only mention vaginal applications of hot water, glycerine, iodine, ichthyol, etc., for many of you use them successfully, though few think it worth while to emphasize the good results by reporting them. If these simple remedies fail, you should at once procure an electric outfit and learn the technic of its proper application. This will be troublesome, of course, and you may yearn to remove the parts at once by an abdominal or pelvic section, even though your list be well up in the hundreds, but strict professional duty requires that we either do what is best for the patient, or else leave her alone.

The young wife of a prominent dentist consulted me some years ago for a peristent menorrhalgia and intermenstrual pain. The menorrhalgia dated back into her recent girlhood, and before marriage she had been subjected to dilatation of the cervix under ether by a skilled surgeon, with the only favorable result of one period without pain, but the operation was followed by intermenstrual pains in the region of the left ovary. When seen first by the speaker the cervix showed a stellate laceration and the left ovary was enlarged, prolapsed and tender. She had remained sterile. Vaginal galvanic applications of the positive pole were begun and kept up for a number of months with encouraging results in relieving both kinds of pain and the tenderness, and when finally it was thought that the ovarian tenderness had subsided sufficiently, the uterus itself received direct attention for the still existing endometritis that had been the primary fault. A few positive galvanic intra-uterine applications were made, small currents being employed because, even at the frequency of once a week, they were followed by pain. These were quickly followed by added relief, and after several months further treatment by both intra-uterine and vaginal applications, she was discharged cured, and only shortly afterwards the first conception occurred.

This patient had distinct ovarian disease when first seen, tossed her bed in agony every month, and had been married and sterile over a year. Removal of the ovaries had of course been advised. She now has painless periods and is the mother of a fine boy a year and a half old.

A case somewhat similar in origin and conditions present was that of Miss B., aged 19, a handsome girl, kindly referred by

Dr. W. G. Leaman of West Philadelphia. She had a family history of consumption and a personal history of delayed and irregular puberty, which finally developed intense menorrhspasms that put her in bed a week in each month in a condition of convulsed incoherence from the intense pain. Before seeing Dr. Leaman she had been dilated in hospital by one practitioner; had worn a stem pessary for a year, inserted by another; and had been advised by still another that the only cure was a removal of the ovaries. At this juncture Dr. Leaman was called in, and being loath to have the patient wrecked between the two alternatives of asexualization or the opium habit, he turned her over to the speaker for special attention. The examination showed the uterus slightly anteflexed, enlarged, and the left ovarian region boggy and tender. There was no material amount of leucorrhea.

The treatment was begun August 23, 1898, and terminated in February, 1899, covering six months, though for a portion of this time it was disultory. It consisted almost entirely of vaginal applications of galvanic and faradic currents, positive, though at one time several intra-uterine applications were made, which caused additional pain. To-day, almost a year since the cessation of the treatment, the patient remains in good health, having had but one painful period in over a year, and this one caused by exposure to cold and wet.

Not to go into further detail in this matter of the saving of ovaries, I must testify to the special usefulness of electricity in the cure of endometritis, prolapse of the uterus, and relaxation of the uterine supports, not caused by laceration of the pelvic floor. Even post-gonorrheal endometritis may be cured by the use of mild applications of electro-mercuric sterilization by a method which is a miniature example of the writer's process for the treatment of cancer, leaving for major surgery only those cases of chronic extension of this infection to the tubes.

Finally, it is our duty to be scrupulously conscientious in laying before our patients, who are suffering from benign fibrous tumors of the uterus, the exact status of conservative electric treatment of these cases, giving them the option of immediate operation and almost immediate cure with its probable risks and certain result in unsexing, on the one hand, and on the other hand the option of a long and somewhat tedious treatment that is absolutely harmless in proper cases, and certain to produce a shrinkage and symptomatic cure in ninety per cent. of the cases, including actual disappearance of the tumor by absorption in five or six per cent. of the cases, while those tumors not helped will not be harmed. With these facts properly stated the patient's choice will be intelligent, and governed by the circumstances in which she is placed.

I had intended to give some recent instances of fibroids treated by the Apostoli method, but as this paper is already longer than it should be, I will reserve these notes for a future communication.

*1636 Walnut St.*

